# Pulmonary and cerebral paracoccidioidomycosis

**DOI:** 10.1590/0037-8682-0188-2022

**Published:** 2022-09-19

**Authors:** Matheus Garcia Lago Machado, Rosana Souza Rodrigues, Edson Marchiori

**Affiliations:** 1 Universidade Federal do Rio de Janeiro, Rio de Janeiro, RJ, Brasil.; 2 Instituto D’Or de Pesquisa e Ensino, Rio de Janeiro, RJ, Brasil.

**FIGURE 1:**
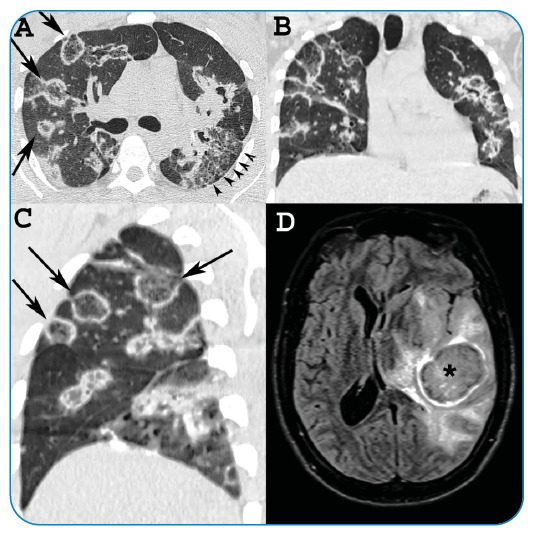
**(A)** Axial, **(B)** coronal, and **(C)** sagittal chest computed tomography images showing multiple round focal areas of ground-glass opacity surrounded by rings of consolidation (reversed halo sign) (arrows). Moreover reticulation is noted on the left side (arrowheads). **(D)** Brain magnetic resonance imaging revealed a large parietotemporal lesion in the left hemisphere (asterisk) with peripheral postcontrast enhancement.

A 39-year-old man presented with a two-month history of holocranial headache, fever, dry cough, and weight loss of 12 kg. Physical examination revealed confusion, gait disturbance, bilateral cervical lymphadenopathy, and amygdala ulceration. Chest computed tomography showed multiple round focal areas of ground-glass opacity surrounded by nearly complete rings of consolidation (reversed halo sign; Figure 1A-C). Brain magnetic resonance imaging revealed a large parietotemporal lesion in the left hemisphere with peripheral postcontrast enhancement (Figure 1D). Lumbar puncture and amygdala swab culture revealed *Paracoccidioides spp*. infection. The patient was treated with intravenous sulfamethoxazole-trimethoprim (400 + 80 mg) and liposomal amphotericin B (300 mg/day). His symptoms regressed completely, and he was discharged 90 days after admission with a prescription for sulfamethoxazole-trimethoprim (400 + 80 mg daily).

Paracoccidioidomycosis (PCM) is caused by the dimorphic fungus *Paracoccidioides brasiliensis,* and its chronic form may progress to severe pulmonary involvement. Its definitive diagnosis should be based on fungal element detection by microscopic examination of fresh clinical specimens or biopsy material, which can be complemented by culturing and isolating the fungus^1^ ^-^ ^3^.

The main pulmonary computed tomography findings in PCM are ground-glass opacities, consolidations, nodules, masses, cavitations, and fibrotic lesions, frequently in combination. A reversed halo sign was observed in approximately 10% of patients with active infection^3^. On T1-and T2-weighted magnetic resonance imaging, brain PCM present variable hypo- or hyperintense signals with annular impregnation after contrast injection and perilesional edema^1^. Imaging evaluation is essential for differential diagnosis and to direct the initial patient care^2^. 
